# Global and temporal shifts in the cultured-based bacteriology of otitis media with effusion: a systematic review and meta-analysis

**DOI:** 10.3389/fpubh.2026.1909909

**Published:** 2026-10-15

**Authors:** Hye Ok Kim, Yeon Ju Oh, Seung Hyung Lee, Jae Min Lee, Youn-Jung Kim, Manish Kumar Singh, Sung Soo Kim, Jin Woo Choi, Seung Geun Yeo

**Affiliations:** 1 Department of Medicine, College of Medicine, Kyung Hee University, Seoul, Republic of Korea; 2 Clinical Research Institute, Kyung Hee University Medical Center, Seoul, Republic of Korea; 3 Department of Otorhinolaryngology – Head and Neck Surgery, College of Medicine, Medical Center, Kyung Hee University, Seoul, Republic of Korea; 4 College of Nursing Science, Kyung Hee University, Seoul, Republic of Korea; 5 Department of Biochemistry and Molecular Biology, College of Medicine, Kyung Hee University, Seoul, Republic of Korea; 6 Department of Pharmacology, College of Pharmacy, Kyung Hee University, Seoul, Republic of Korea

**Keywords:** antibiotic stewardship, antimicrobial resistance, bacterial pathogens, middle ear microbiology, otitis media with effusion

## Abstract

**Objective:**

This study systematically synthesized geographic variation and calendar-period differences in culture-based bacteriology of OME to examine their implications for child hearing health, pathogen surveillance, and antimicrobial stewardship.

**Methods:**

PubMed, Embase, and Cochrane Library were searched for studies published from Jan 1980 to Dec 2025 that reported the results of bacterial culture from patients with OME. Studies were eligible if they included human subjects with otitis media with effusion (OME) and reported the prevalence of bacteria. Studies were excluded if they were unrelated to the review objective or eligibility criteria, duplicate records, non-original reports, non-human studies, studies lacking basic demographic information, studies with insufficient data, or studies not eligible for quantitative synthesis. Three reviewers screened the articles and extracted data. Finally, 22 studies were included (North America: *n* = 4, Europe: *n* = 9, Asia: *n* = 7, Oceania: *n* = 1, Middle East: *n* = 1). Culture positivity was meta-analyzed using culture-positive specimens as the numerator and all cultured specimens as the denominator. Organism-specific proportions were meta-analyzed using culture-positive specimens as the denominator. Raw bacterial isolate counts were summarized separately and were not used as denominators for the meta-analysis.

**Results:**

The random-effects pooled culture-positivity proportion was 43.4% (95% CI, 35.4–51.8%). Among culture-positive specimens, the pooled organism-specific proportions were 26.4% for *H. influenzae*, 26.1% for CoNS, 16.0% for MSSA, 14.2% for *S. pneumoniae*, and 13.1% for MRSA. In North American studies, CoNS and *H. influenzae* showed comparable pooled proportions, whereas *H. influenzae* predominated in European studies and CoNS predominated in Asian studies. Studies conducted from 2000 onward showed higher pooled proportions of CoNS and MSSA and lower pooled proportions of *H. influenzae* and *S. pneumoniae* than earlier studies.

**Conclusion:**

The bacteriological profile of OME varied meaningfully by region and period. These findings support strengthened pathogen surveillance and antimicrobial stewardship in the context of childhood hearing health, rather than a single universal empirical management approach.

**Systematic review registration:**

The protocol was registered in the International Prospective Register of Systematic Reviews, https://www.crd.york.ac.uk/PROSPERO/home, PROSPERO, CRD420261291723.

## Introduction

Otitis media (OM) encompasses a spectrum of inflammatory diseases of the middle ear that are common in children, and includes acute otitis media (AOM), otitis media with effusion (OME, also known as “glue ear”), and chronic suppurative otitis media (CSOM) (1). OME is defined by the presence of a middle-ear fluid (MEF) without the rapid onset of signs of an acute infection or other symptoms (fever, ear pain, or bulging tympanic membrane) (2). OME is categorized as acute OME if the effusion lasts less than 3 months, and otherwise as chronic OME. OME is extremely common in childhood, and up to 80% of children experience at least one episode by the age of 4 years. Because MEF dampens vibrations of the tympanic membrane and impairs sound conduction, OME is also the leading cause of acquired hearing loss in children. Most patients experience spontaneous resolution of effusions within 3 months; however, about 30–40% of patients experience recurrence. Persistent OME often has no symptoms other than hearing impairment. Nevertheless, conductive hearing loss during childhood can adversely affect the development of speech, language, and cognition, and thereby impair quality of life due to learning difficulties and behavioral problems (3). Although OME is often approached as a routine otologic condition, its public health relevance lies in its contribution to hearing loss during early childhood, when auditory input is essential for speech, language, learning, and social development.

Bacterial pathogens are a main cause of OME and AOM. The MEF of patients with OME and AOM often harbor the same pathogens, although OME typically has a lower bacterial load. The three classic “otopathogens” most commonly identified in OME [*Streptococcus pneumoniae*, nontypeable *Haemophilus influenzae* (NTHi), and *Moraxella catarrhalis*] account most positive results in cultures of MEF from these patients. Notably, these same three species are responsible for approximately 80% of AOM episodes, but their relative prevalence can differ in these two types of OM (4).

Several other bacteria can also cause OME. For example, *Staphylococcus aureus* has been cultured from some MEF samples, including methicillin-susceptible (*MSSA*) and methicillin-resistant (*MRSA*) strains. Recent reports from certain regions have also noted high rates of *MRSA* in patients with chronic OME, suggesting possible nosocomial infection or antibiotic-driven selection of resistant strains. *Coagulase-negative staphylococci* (*CoNS*), such as *Staphylococcus epidermidis*, were also isolated in some OME effusions. Although earlier studies attributed these findings to contamination, their consistent recovery—especially in patients with post-tympanostomy otorrhea or longstanding OME—raises the possibility that biofilms of this species contribute to chronic inflammation (5). Some researchers reported *Streptococcus pyogenes* and *Pseudomonas aeruginosa* in OME samples (6). These diverse microbiological findings likely reflect differences in patient populations and detection techniques among studies. Indeed, there is evidence of differences in the pathogens in OME samples in patients who live in different regions and in patients studied at different times, underscoring the need for a region-specific and up-to-date analysis of these data (4, 6).

Several recent developments point to the need for an updated systematic synthesis of OME bacteriology. First, widespread implementation of pneumococcal conjugate vaccines (PCVs) has altered pneumococcal carriage and serotype ecology (7). Population-based evidence suggests that PCVs can influence the epidemiology of OME in certain settings (8, 9). Second, antimicrobial resistance continues to increase, and resistance patterns are especially relevant for patients with recurrent OME, perioperative conditions, or treatment failure (1). Third, the expanding global literature and increasing use of PCR and other sensitive detection methods strengthen the rationale for a contemporary synthesis that focuses on geographic and temporal variability.

The objective of this systematic review and meta-analysis was to synthesize regional distributions and temporal changes of cultured-based bacteria in OME and to assess the public health implications of these patterns for pediatric hearing health, temporal and regional pathogen surveillance, and antimicrobial stewardship. This study aims to inform surveillance priorities, stewardship efforts, and prevention-oriented public health strategies in settings where local microbiological evidence remains limited.

## Methods

### Data sources and searches strategy

This systematic review and meta-analysis was conducted in accordance with PRISMA guidelines (Figure 1) and the protocol was registered in the International Prospective Register of Systematic Reviews (PROSPERO, CRD420261291723). The PubMed, Embase, and Cochrane Library databases were searched for relevant studies that were published from January 1, 1980 to December 31, 2025 using the following database-specific Boolean search strings: PubMed, ((“otitis media with effusion”[MeSH]) AND (“bacteria”[tiab] OR “bacteriology”[Mesh])); Embase, ((“otitis media with effusion”:ti,ab) AND (bacter*:ti,ab OR microbiolog*:ti,ab)); and Cochrane Library, ((“otitis media with effusion”):ti,ab,kw AND (bacteriolog* OR microbiolog*):ti,ab,kw)) (Supplementary Table 1).

**Figure 1:**
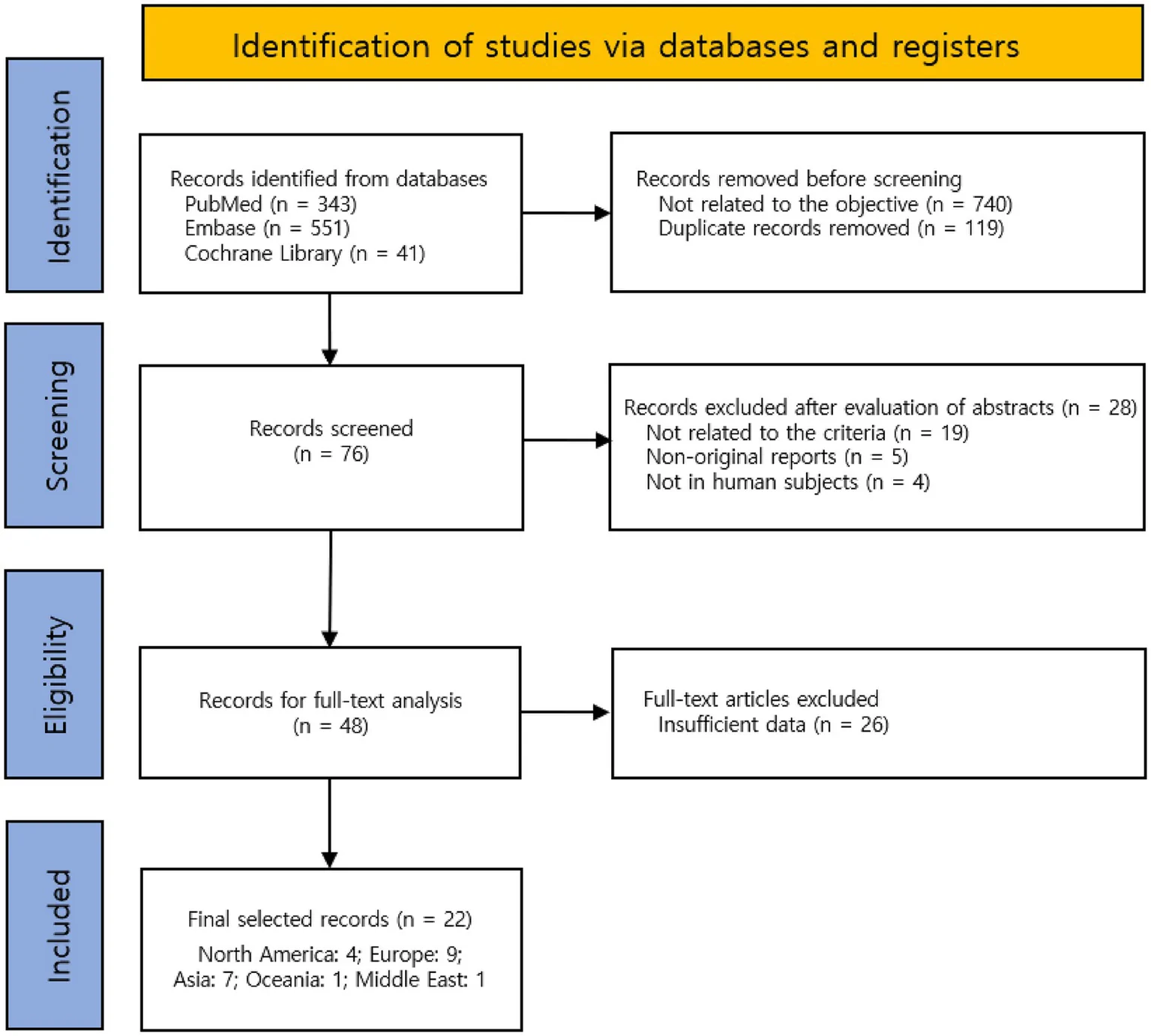
PRISMA flow diagram of the literature search strategy.

### Study selection

Studies were eligible if they included human subjects with otitis media with effusion (OME) and reported the prevalence of bacteria in adenoid culture, mucosal biopsy, and middle ear fluid (MEF) samples obtained via clinical perforation, such as myringotomy or tympanocentesis. Studies were eligible if they reported culture-based bacteriological findings in OME and provided extractable numerator and denominator data for culture positivity and/or organism-specific specimen-level detection. Studies were excluded if they were unrelated to the review objective or eligibility criteria, duplicate records, non-original reports, non-human studies, studies reporting data already included in an earlier publication, studies based on spontaneous tympanic membrane rupture, studies analyzing otorrhea exclusively from tympanostomy tubes, studies lacking basic demographic information, studies with insufficient data, or studies not eligible for quantitative synthesis.

A total of 935 records were identified through database searching, including 343 from PubMed, 551 from Embase, and 41 from the Cochrane Library. Before screening, 740 records were removed because they were not related to the objective and 119 duplicate records were removed. The remaining 76 records underwent screening. Of these, 28 records were excluded after abstract evaluation because they were not related to the criteria, were non-original reports, or were not in human subjects. Subsequently, 48 records underwent full-text analysis. Of these, 26 full-text articles were excluded because they contained insufficient data. Consequently, 22 records were finally selected (4 from the Americas, 9 from Europe, 7 from Asia, 1 from Oceania, and 1 from Middle East).

### Data extraction

Three authors (H. O. K., Y. J. O., and S. G. Y.) independently examined all 25 included studies. For each study, they reviewed the abstract, identified additional potentially related studies by review of the references, read the full text, confirmed eligibility, and extracted the data for meta-analysis. The extracted variables included patient age, country or region, study period, number and type of cultured specimens, number of culture-positive specimens, organism-specific positive-specimen counts, and reported bacterial isolate counts. Whether specimens were obtained unilaterally or bilaterally was also recorded when reported. Missing information was recorded as ‘not documented’. Missing values were marked as missing. In cases where OME data were part of a broader study, only data related to OME were extracted. All full-text articles selected for inclusion were reviewed in detail, and any disagreements among the three reviewers regarding eligibility were resolved through discussion. Reviewers did not evaluate their own publications.

### Risk of bias assessment

At the time of data extraction, the methodological quality and risk of bias of each report was assessed using the Joanna Briggs Institute Prevalence Critical Appraisal Tool (10). The total score of each study was a percentage calculated as the number of positive responses to nine criteria, each of which had a possible response of “yes” (one point) or “no” or “unclear” (zero points). All nine criteria were evaluable in all included studies. Each study was then categorized as having a “high risk of bias” (≤33%), “moderate risk of bias” (34–66%), or “low risk of bias” (>66%) (11). Two reviewers independently confirmed that each study was scored correctly. Any ambiguity or disagreement was resolved by consensus or by consulting the third reviewer (Supplementary Table 2).

### Data synthesis and analysis

Only studies providing extractable numerator and denominator data for a given outcome were included in the corresponding quantitative synthesis. Three levels of data were distinguished: cultured specimens, culture-positive specimens, and bacterial isolates. Patient counts and ear counts were recorded as study characteristics but were not treated as interchangeable with specimen counts. For the quantitative microbiological syntheses, the analytic unit was standardized to the cultured specimen whenever a specimen-level numerator and denominator could be reconstructed from the original report. Culture positivity was defined as the number of culture-positive specimens divided by the total number of specimens cultured. For organism-specific analyses, the numerator was the number of culture-positive specimens yielding the organism of interest, and the denominator was the number of culture-positive specimens in studies reporting that organism. When multiple bacterial species were recovered from the same specimen, the specimen was counted once in the culture-positivity analysis but contributed once to the numerator for each organism detected; thus, organism-specific proportions were not mutually exclusive and were not expected to sum to 100%. Multiple isolates of the same species from a single specimen were counted once in specimen-based organism-specific analyses when specimen-level information was available. Studies that reported only isolate counts without a reconstructable specimen-level denominator were retained for descriptive isolate-level summaries but were excluded from the corresponding specimen-based meta-analysis. Total bacterial isolate counts were therefore used only to describe isolate composition and were not used as denominators for pooled organism-specific proportions. Data were tabulated using Microsoft Excel and analyzed using R version 4.5.1. Meta-analyses of proportions were performed using the metaprop function in the meta package, and forest plots were generated using the forest function. For each outcome, pooled proportions and 95% confidence intervals (CIs) were calculated using fixed-effect and random-effects models. Statistical heterogeneity was quantified using the I^2^ and τ^2^ statistics. Because between-study heterogeneity was substantial for most outcomes (median *I*^2^ > 80%), random-effects estimates were used for the primary interpretation.

For the temporal subgroup analysis, studies were classified according to the calendar period in which participant enrollment and specimen collection were conducted, rather than the year of publication. Studies in which data collection was completed by December 31, 1999 were classified as pre-2000, whereas studies in which data collection began on or after January 1, 2000 were classified as 2000-onward. Studies whose data-collection periods crossed the year 2000 were excluded from the primary temporal subgroup analysis unless year-specific numerator and denominator data were separately extractable. Studies for which the data-collection period could not be determined were also excluded from the temporal analysis. As a sensitivity analysis, studies spanning the year 2000 were additionally assigned according to the midpoint of their reported data-collection period, and the temporal subgroup analyses were repeated to assess whether the direction and magnitude of the pooled estimates were robust to the classification rule. This sensitivity analysis was conducted *post hoc* to assess the robustness of the findings to uncertainty in the classification of studies spanning the calendar cutoff. As a *post hoc* assessment of potential cohort overlap, Korean studies were compared with respect to study period, institution or study center, author/research network, patient population, clinical procedure, and specimen source. Because participant-level linkage was unavailable, overlap was classified as possible or probable rather than confirmed. Sensitivity analyses were then performed by excluding all Korean studies and by applying a conservative exclusion strategy for potentially overlapping Korean cohorts. Potential publication bias was assessed by visual inspection of funnel plots (Supplementary Table 3).

## Results

The literature search led to the initial identification of 935 studies, 22 of which met the inclusion criteria for the qualitative synthesis (12–36) (Figure 1 and Table 1). The 22 included studies contributed 5,682 cultured OME-related specimens to the culture-positivity analysis, of which 2,002 were culture-positive. The random-effects pooled culture-positivity proportion was 43.4% (95% CI, 35.4–51.8%). Separately, 1,926 organism-specific isolate records were reported in the current descriptive dataset (Supplementary Table 4). Most studies focused on pediatric patients, in that they mainly enrolled infants and young children (common upper age limit: ~12 years), although a few investigations studied adolescents or adults; one study included a patient who was 61-years-old and another study included a patient who was 91-years-old. There were four studies from North America (all in the United States), nine from various European countries, seven from Asia (five from South Korea, and one each from China and Thailand), and one each from Oceania (Australia) and the Middle East (Iran). The sampling methods were predominantly MEF cultures during tympanostomy or myringotomy, although four studies analyzed adenoid tissues or middle ear mucosal biopsy specimens (Table 1).

**Table 1:** Characteristics of the 22 included studies and analytic denominators in the cultured-based bacteriological analysis of OME.

Continent country	Study onset (year)	Study end (year)	Youngest patient (months)	Oldest patient (months)	No. of cultured specimens	Dominant bacteria^†^	Technique	References
North America								
USA	ND	ND	48	132	50	HI, SP, MSSA	MEF culture	(12)
USA	2012	2013	6	144	84	CoNS, HI, MC	MEF culture	(13)
USA	2001	2002	6	120	446	HI, MC, SP	MEF culture	(14)
USA	1978	1980	0	144	612	HI, SP, CoNS	MEF culture	(15)
Europe								
Poland	2018	2020	6	144	99	HI, SP, SA	MEF culture	(17)
Belgium	ND	ND	12	79	34	HI, SP, MC	MEF culture, Adenoid culture	(19)
Finland	1999	2000	7	111	62	MC, HI, SP, MSSA	MEF culture	(20)
Sweden	2006	2008	12	96	35	HI, MC, SP	MEF culture	(21)
England	1986	1987	24	240	259	HI, SP, MSSA	MEF culture	(23)
Finland	1993	1994	5	144	165	SP, HI, MC	MEF culture	(24)
Finland	ND	1999	19	90	18	CoNS, SP, MSSA	Mucosal biopsy	(25)
England	ND	1989	12	120	102	HI, CoNS, SP	MEF culture	(26)
Germany	1985	1986	25	163	50	CoNS, HI, MSSA, SP	MEF culture	(27)
Asia								
Korea	2010	2018	10	204	278	CoNS, MRSA, SA, SP, HI	MEF culture	(28)
Korea	2003	2012	12	216	847	CoNS MSSA MRSA	MEF culture	(29)
Korea	2004	2008	24	84	356	CoNS, MRSA, MSSA	MEF culture	(30)
Korea	2000	2020	6	349	1,335	CoNS, MSSA, SP	MEF culture	(31)
Korea	2001	2010	6	1,092	381	CoNS, MSSA, SP	MEF culture	(32)
China	2013	2020	0	192	311	MSSA, MC, SP	Adenoid culture	(33)
Thailand	2005	2018	12	120	78	HI, MSSA, SP	Adenoid culture	(34)
Oceania								
Australia	2011	2012	9	36	38	HI, SP, MSSA	MEF culture	(35)
Middle East								
Iran	2014	2015	24	144	42	PA, SP, MSSA	MEF culture	(36)

^†^CoNS, Coagulase-negative staphylococci; HI, *Haemophilus influenzae*; MSSA, Methicillin-susceptible *S. aureus*; SP, *Streptococcus pneumoniae*; MC, *Moraxella catarrhalis*; MRSA, Methicillin-resistant *S. aureus*; PA, *Pseudomonas aeruginosa*; ND, Not Documented.

In the descriptive isolate-level dataset (*n* = 1,926 isolate records), *S. aureus* accounted for 557 records (28.9%), comprising 329 MSSA records (17.1%) and 228 MRSA records (11.8%). CoNS accounted for 407 (21.1%), *H. influenzae* for 328 (17.0%), *S. pneumoniae* for 250 (13.0%), *M. catarrhalis* for 119 (6.2%), *P. aeruginosa* for 90 (4.7%), and other bacteria for 175 (9.1%) (Supplementary Table 4). These percentages describe raw isolate composition and are not pooled proportion estimates.

There were notable geographical differences in culture positivity and in the most prevalent species (Figure 2). The pooled culture-positivity proportion (culture-positive specimens/all cultured specimens) was higher in North America (0.487, 95% CI: 0.243–0.738) than Europe (0.397, 95% CI: 0.323–0.476) and Asia (0.475, 95% CI: 0.246–0.715). Among culture-positive specimens, *H. influenzae* had the highest pooled organism-specific proportion in Europe (34.7%), followed by CoNS (18.7%), *S. pneumoniae* (18.4%), and *M. catarrhalis* (12.4%). In North America, CoNS (30.6%) and *H. influenzae* (30.1%) showed comparable pooled organism-specific proportion, followed by *M. catarrhalis* (13.3%) and *S. pneumoniae* (12.5%). The Asian studies showed a different profile; however, five of the seven Asian studies were from South Korea. CoNS had the highest pooled organism-specific proportion (31.8%), followed by MSSA (23.0%) and MRSA (17.6%); *S. pneumoniae* had a pooled organism-specific proportion of 11.5% and *H. influenzae* and *M. catarrhalis* were very rare (Figure 3). In a *post hoc* sensitivity analysis excluding all Korean studies, the pooled estimates were 55.8% for culture positivity, 36.5% for *H. influenzae*, 17.9% for CoNS, 19.7% for *S. pneumoniae*, 15.4% for MSSA, and 14.0% for MRSA. Compared with the primary analysis, the largest shifts among these major organisms were observed for *H. influenzae* and CoNS, whereas the MSSA and MRSA point estimates changed only modestly. A separate conservative analysis addressing potentially overlapping Korean cohorts yielded less precise estimates for several outcomes because of the reduced number of independent study-level estimates (Supplementary Table 5). In the primary temporal analysis based on study conduct period, studies completed before 2000 were compared with studies initiated in 2000 or later. Studies spanning the cutoff and those with undetermined study periods were excluded from this analysis. The pooled culture-positivity proportion increased from 38.3 to 42.7%. Among culture-positive specimens, the pooled organism-specific proportion of *H. influenzae* decreased from 30.2 to 23.7%, *S. pneumoniae* from 17.9 to 13.6%, whereas CoNS increased from 17.6 to 34.8% and MSSA from 7.1 to 23.2%. The pooled organism-specific proportion of *M. catarrhalis* was low during both periods, but was also greater in the more recent period (4.6% vs. 8.4%) (Figure 4).

**Figure 2:**
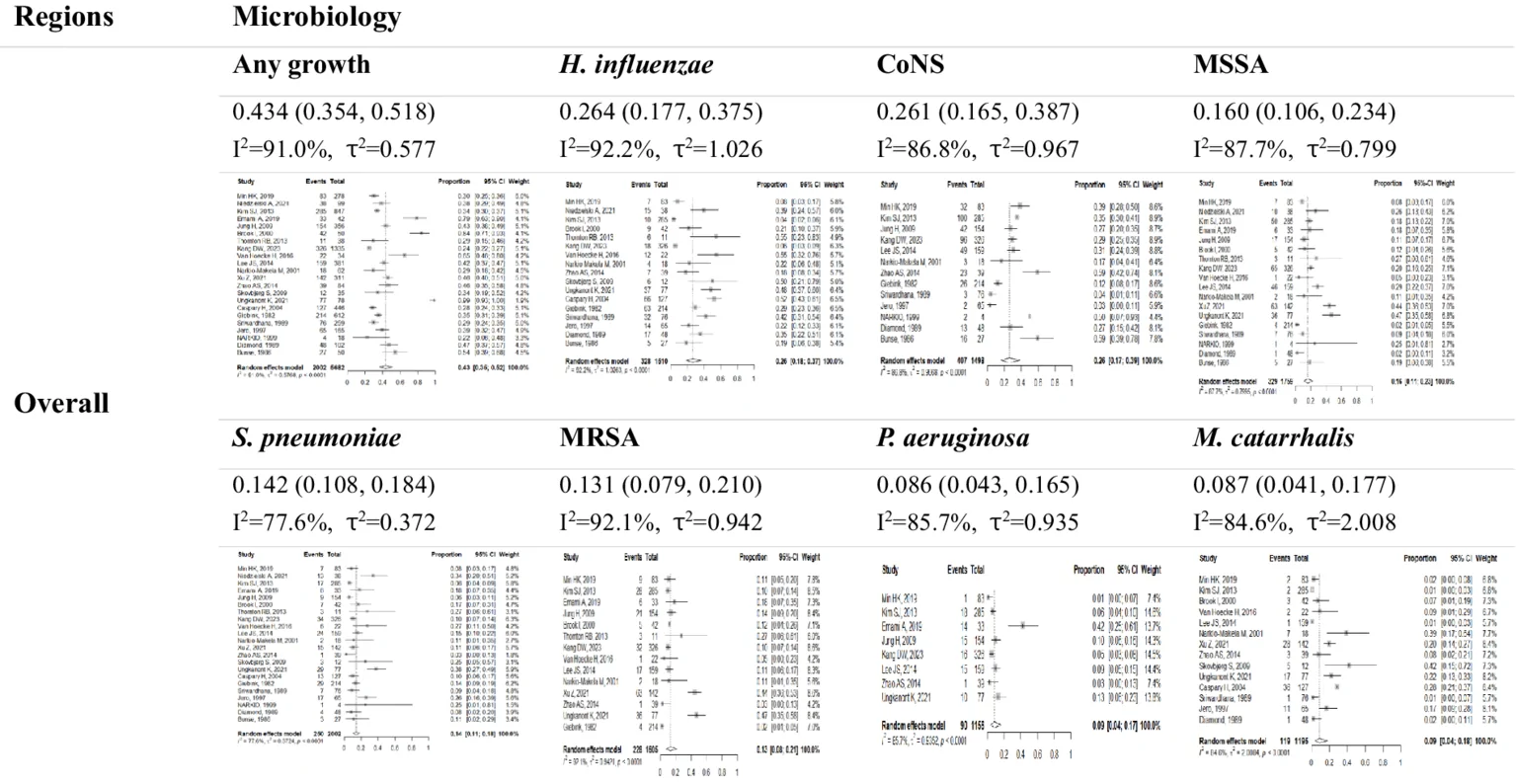
Random-effects pooled estimates of culture positivity and organism-specific proportions in OME (overall). *Culture positivity was calculated as the number of culture-positive specimens divided by the total number of cultured specimens. Organism-specific proportions were calculated as the number of culture-positive specimens yielding the organism of interest divided by the number of culture-positive specimens in the contributing studies. A culture-positive specimen containing more than one bacterial species could contribute to the numerator of more than one organism-specific analysis; therefore, organism-specific proportions are not mutually exclusive and are not expected to sum to 100%. Raw isolate frequencies were summarized separately and were not used as denominators for the pooled meta-analyses. **For the “Any growth” analysis, Events indicates culture-positive specimens and Total indicates all cultured specimens. For organism-specific analyses, Events indicates specimens positive for the specified organism and Total indicates culture-positive specimens in the studies contributing to that analysis.

**Figure 3:**
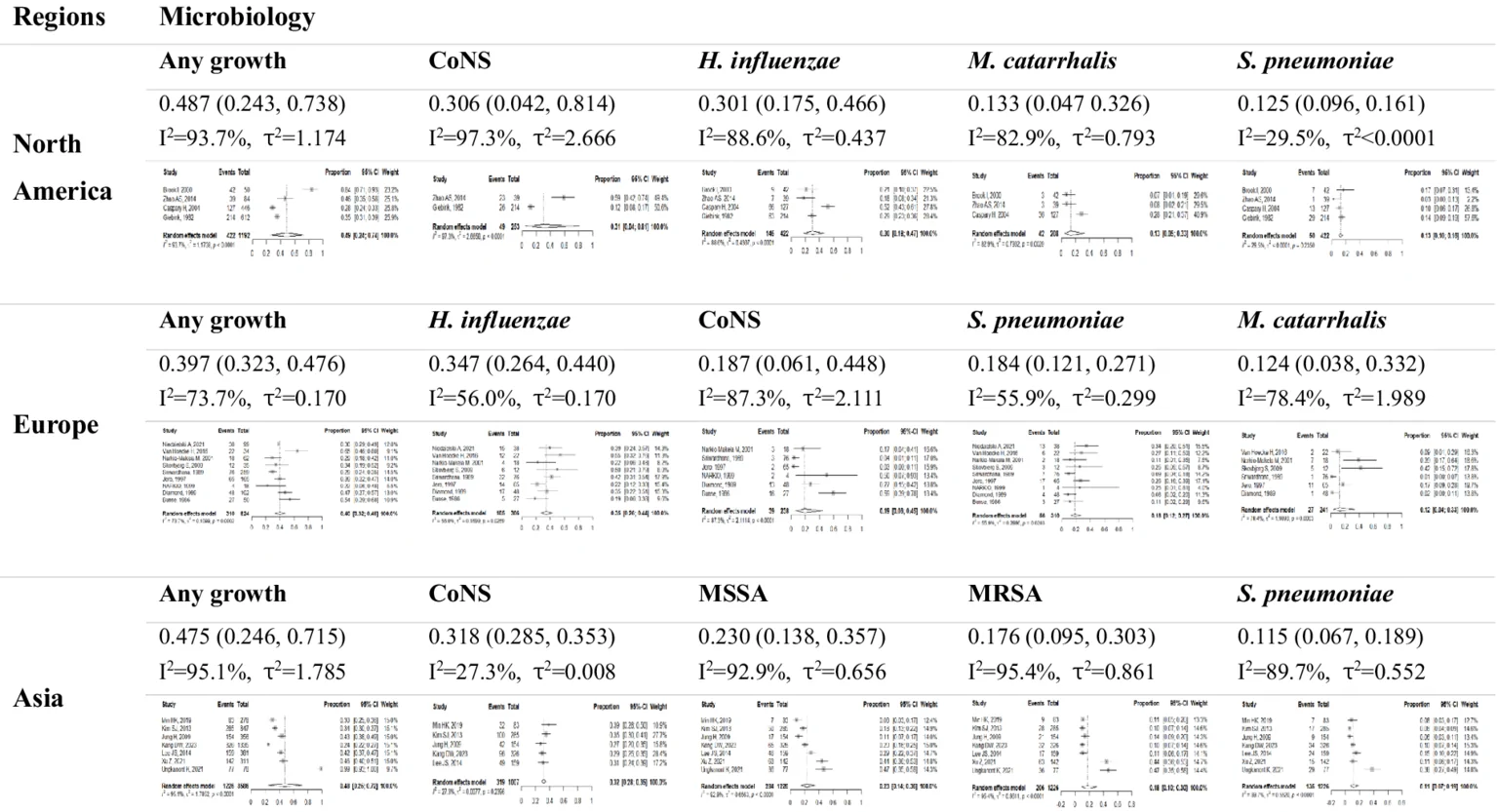
Regional random-effects pooled estimates of culture positivity and organism-specific proportions in OME.

**Figure 4:**
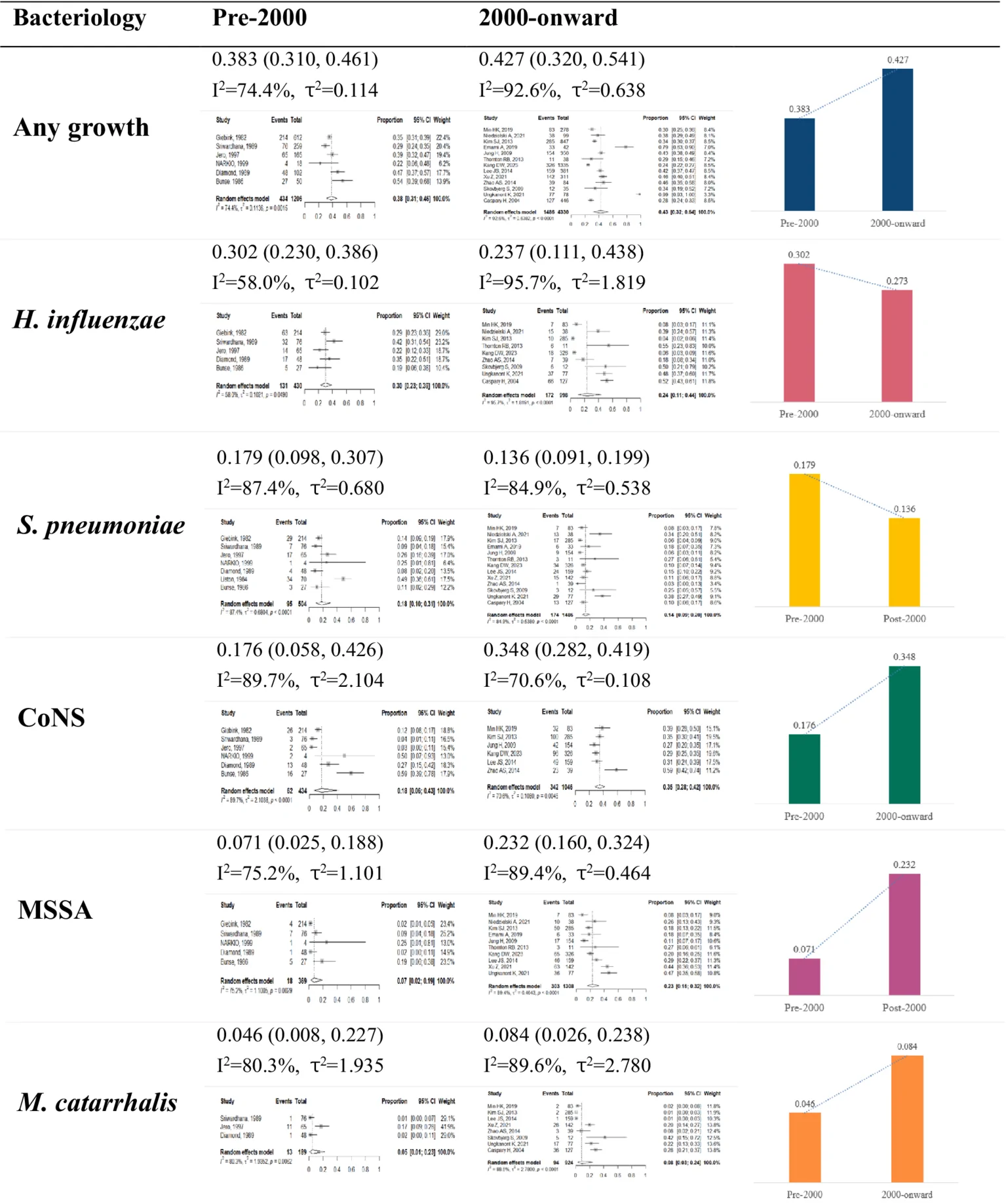
Temporal comparison of pooled culture positivity and organism-specific proportions according to study conduct period.

## Discussion

The 22 included studies contributed 5,682 cultured specimens to the culture-positivity analysis. The random-effects pooled culture-positivity proportion was 43.4%. In the specimen-based organism-specific meta-analysis, *H. influenzae* had the largest pooled proportion among culture-positive specimens (26.4%), followed by CoNS (26.1%) and MSSA (16.0%). There were also notable regional differences in pooled organism-specific proportions. *H. influenzae* had higher pooled proportions in European studies, whereas several Asian studies, predominantly from South Korea, showed greater representation of staphylococci. In North America, CoNS and *H. influenzae* showed comparable pooled organism-specific proportion. Calendar-period analyses also showed lower pooled proportions of *H. influenzae and S. pneumoniae* and higher pooled proportions of CoNS and MSSA in studies conducted from 2000-onward. However, since a correlation between geographic region and study period was observed in the existing literature, these differences across periods should not be interpreted as evidence of a global temporal trend independent of geography.

Our meta-analysis identified pronounced geographic heterogeneity in the bacteriology of OME. North America and Europe were dominated by the classical OM bacteria (*H. influenzae*, *S. pneumoniae*, and *M. catarrhalis*), whereas Asia had a predominance of staphylococci. This typical Western pattern, wherein these three otopathogens accounted for most OME infections, closely mirrors their predominance in AOM (4). This similarity in the causative pathogens in North America and Europe underscores the continuing ubiquity of these classic middle-ear pathogens in Western pediatric populations. In contrast, the seven Asian studies (5 from South Korea and 1 each from China and Thailand) had markedly different microbiological profiles. Asian studies showed higher pooled organism-specific proportions of staphylococci among culture-positive specimens than of the classical otopathogens (28–32). These pronounced regional differences may reflect multiple factors, including differences in sampling methods, prior antimicrobial exposure, antimicrobial resistance patterns, vaccination programs, patient characteristics, and prior otologic interventions. Accordingly, regional variation in OME bacteriology should not be interpreted solely as a geographic effect, because differences in clinical case mix, treatment history, and local antimicrobial ecology may also contribute to the observed patterns.

Our comparison of studies conducted before 2000 and from 2000 onward identified differences between calendar-period strata. These comparisons are descriptive because geographic composition differed between the two periods and therefore do not establish an independent temporal effect. Although the overall culture positivity increased from 38.3% (before 2000) to 42.7% (2000-onward), the composition of bacteria shifted in tandem with broader epidemiologic and clinical trends. Thus, the classical otopathogens appeared to become somewhat less dominant during recent years. Specifically, the pooled proportion of *H. influenzae* among culture-positive specimens decreased from 30.2% (before 2000) to 23.7% (2000-onward), and *S. pneumoniae* also showed a slight decline (17.9–13.6%). These differences coincided with major changes in otitis media epidemiology and clinical practice, including the progressive introduction of pneumococcal conjugate vaccines (PCV) in many countries. After the introduction of PCV7, *S. pneumoniae* infections fell and NTHi became the predominant cause of OM in some countries (7). A recent population-based study in Vietnam found that the introduction of PCV10 led to a significant reduction in the prevalence of OME in young children (8). However, the year 2000 was used in the present analysis as a calendar-period cutoff and not as a direct measure of PCV exposure. PCV implementation, vaccine formulations, coverage, antimicrobial use, diagnostic practices, and microbiological methods varied substantially across countries and over time. These findings should therefore be considered temporal associations rather than evidence of a causal vaccine effect.

Studies conducted from 2000 onwards showed higher pooled proportions of CoNS (34.8% vs. 17.6%) and MSSA (23.2% vs. 7.1%) compared to studies conducted prior to 2000. This suggests a shifting pattern in the nature of OME and aligns with research indicating that *S. aureus* is becoming an increasingly common cause of OME in multiple regions (30). However, these between-period differences should not be interpreted as demonstrating a global increase over time, because recent studies were disproportionately represented by Asian, particularly South Korean, cohorts. Several factors may explain these findings. One is the increased awareness and detection of these pathogens. Previously, *CoNS* was often dismissed as a skin contaminant (27), but more recent research considers this pathogen capable of forming biofilms and potentially contributing to the pathology of chronic OME (17). Prior otologic interventions and treatment exposure may represent additional sources of between-study heterogeneity. Evidence from chronic ear disease suggests that operated and non-operated patients can have different microbial distributions and antimicrobial resistance profiles. Aliyeva and Sari reported that, among patients with chronic otorrhea, *P. aeruginosa* was more frequently identified in non-operated patients, whereas *E. coli* was more frequent in operated patients (37); resistance in *S. aureus* was also observed only in the operated group. Although these findings were derived from chronic otorrhea rather than OME and therefore cannot be directly extrapolated to our study population, they support consideration of prior surgical intervention and treatment exposure when interpreting middle-ear bacteriological findings. This possibility is also supported by OME-specific studies included in our review. Sriwardhana et al. reported significantly fewer culture-positive middle-ear effusions among children who had received antibiotics before surgery (23), whereas Jero and Karma found a lower frequency of major otopathogens among previously adenotomized children than among non-adenotomized children (24). Accordingly, the regional and temporal differences observed in the present meta-analysis may partly reflect differences in prior otologic procedures, antimicrobial exposure, referral patterns, and local resistance ecology, in addition to vaccination, sampling methods, and other secular changes. Interpretation of pathogen distributions should therefore take patient treatment history and local antimicrobial susceptibility data into account, particularly when antimicrobial therapy is clinically indicated.

## Limitations

Despite the rigorous methodology, our systematic review and meta-analysis of OME has several limitations. First, the included studies varied in clinical and methodological characteristics, especially patient demographics and methods for bacterial culture sampling, and this may have biased the pooled estimates. Second, concerns variation in the units of reporting across the primary studies. Some studies reported numbers of patients, others numbers of ears or cultured specimens, and some reported only organism-level isolate counts. We therefore standardized the microbiological meta-analyses at the specimen level whenever an appropriate numerator and denominator could be reconstructed. In addition, some patients contributed specimens from both ears. Because the primary reports generally did not provide sufficient information to model the correlation between bilateral specimens within the same patient, such specimens were analyzed according to the units reported in the original studies. This potential within-patient clustering should be considered when interpreting the precision of the pooled estimates. Studies without reconstructable specimen-level denominators were excluded from the corresponding specimen-based meta-analysis and retained only for descriptive analyses when appropriate. Third, although we analyzed studies conducted in North America, Europe, and Asia, all North American studies were conducted in the United States and five of the seven Asian studies were conducted in South Korea. This geographic concentration limits the generalizability of region-specific estimates and may also contribute to the observed calendar-period differences. Because the number of independent study-level estimates was insufficient within several organism-specific region-by-period strata, we could not reliably perform multivariable meta-regression including both study period and region or formal temporal analyses stratified by region. Residual confounding between calendar period and geography therefore cannot be excluded. Fourth, the statistical heterogeneity was high (*I*^2^ > 80%), indicating a substantial variability in outcomes among studies. Even with the use of a random-effects model and subgroup analysis, this high heterogeneity suggests that the pooled estimates should be interpreted with caution. These limitation concerns the temporal subgroup analysis. The year 2000 was used as a calendar cutoff based on study conduct period and should not be interpreted as a uniform boundary between pre- and post-PCV exposure across countries. PCV introduction and vaccine coverage varied geographically and temporally. Moreover, geographic region and study period were not independent in the available literature, because a substantial proportion of the Asian studies were conducted in the more recent period. Consequently, the observed temporal differences may partly reflect geographic composition, changes in sampling and culture techniques, antimicrobial exposure, or other secular trends rather than a discrete effect of calendar period or vaccination. In addition, prior otologic interventions and antimicrobial treatment histories were not reported in a sufficiently standardized manner across the included studies to permit formal subgroup analysis or adjustment for these factors. We therefore cannot exclude residual confounding related to prior tympanostomy or other otologic procedures, previous antimicrobial exposure, or differences in local antimicrobial susceptibility patterns. Finally, there is a possibility of potential publication bias. Many of the included studies were conducted in specialized settings (e.g., tertiary-care centers where surgical sampling was performed) and often-enrolled patients with severe or chronic OME. This could have led to higher rates of pathogen detection, and inflated our overall estimates of prevalence. These limitations mean that the pooled estimates should be interpreted primarily as surveillance-oriented evidence and hypothesis-generating signals rather than as definitive grounds for universal empirical prescribing recommendations. This caution is particularly important given the geographic concentration of the available studies, the heterogeneity of sampling methods, the reliance on culture-based detection, and the substantial between-study heterogeneity.

## Conclusion

Overall, culture-based OME bacteriology remains dominated by *H. influenzae* and *S. pneumoniae*, but its distribution varies substantially by region and study period. Regionally, CoNS and *H. influenzae* showed comparable pooled proportions in North America, *H. influenzae* predominated in Europe, and CoNS predominated in Asia. Studies conducted from 2000 onward showed lower pooled proportions of *S. pneumoniae* and higher proportions of staphylococci than earlier studies; however, these temporal associations should be interpreted cautiously given substantial between-study heterogeneity (*I*^2^ > 80%), shifts in the geographic composition of the evidence base, variation in pneumococcal vaccine implementation, and differences in microbiological methods. These findings underscore the importance of regionally tailored strategies for prevention and treatment of OME. Integrating country-specific microbiological surveillance with vaccination programs and antimicrobial stewardship may help inform context-specific OME prevention and management strategies. Overall, this review repositions OME to a broader public health issue linked to preventable hearing loss, surveillance capacity, and rational antimicrobial use. More methodologically standardized surveillance will be essential for translating microbiological evidence into equitable prevention and management strategies.

## Data Availability

The original contributions presented in the study are included in the article/Supplementary material, further inquiries can be directed to the corresponding author.

## References

[1] Rosenfeld RM Shin JJ Schwartz SR Coggins R Gagnon L Hackell JM . Clinical practice guideline: otitis media with effusion (update). Otolaryngol Head Neck Surg. (2016) 154:S1–S41. doi: 10.1177/0194599815623467, 26832942

[2] Harmes KM Blackwood RA Burrows HL Cooke JM Harrison RV Passamani PP. Otitis media: diagnosis and treatment. Am Fam Physician. (2013) 88:435–40. 24134083

[3] Searight FT Singh R Peterson DC. Otitis Media With Effusion. StatPearls. Treasure Island, FL: StatPearls Publishing (2026). 30855877

[4] Ngo CC Massa HM Thornton RB Cripps AW. Predominant bacteria detected from the middle ear fluid of children experiencing otitis media: a systematic review. PLoS One. (2016) 11:e0150949. doi: 10.1371/journal.pone.0150949, 26953891 PMC4783106

[5] Ramakrishnan K Sparks RA Berryhill WE. Diagnosis and treatment of otitis media. Am Fam Physician. (2007) 76:1650–8. 18092706

[6] Schilder AGM Chonmaitree T Cripps AW Rosenfeld RM Casselbrant ML Haggard MP . Otitis media. Nat Rev Dis Primers. (2016) 2:16063. doi: 10.1038/nrdp.2016.63 PMC7097351 27604644

[7] Wiertsema SP Kirkham LAS Corscadden KJ Mowe EN Bowman JM Jacoby P . Predominance of nontypeable *Haemophilus influenzae* in children with otitis media following introduction of a 3+0 pneumococcal conjugate vaccine schedule. Vaccine. (2011) 29:5163–70. doi: 10.1016/j.vaccine.2011.05.035, 21621576

[8] Toizumi M Satoh C Quilty BJ Nguyen HAT Madaniyazi L Le LT . Effect of pneumococcal conjugate vaccine on prevalence of otitis media with effusion among children in Vietnam. Vaccine. (2022) 40:5366–75. doi: 10.1016/j.vaccine.2022.07.047, 35934579

[9] Wannarong T Ekpatanaparnich P Boonyasiri A Supapueng O Vathanophas V Tanphaichitr A . Efficacy of pneumococcal vaccine on otitis media: a systematic review and meta-analysis. Otolaryngol Head Neck Surg. (2023) 169:765–79. doi: 10.1002/ohn.327, 36924215

[10] Munn Z Moola S Lisy K Riitano D Tufanaru C. Methodological guidance for systematic reviews of observational epidemiological studies reporting prevalence and cumulative incidence data. Int J Evid Based Healthc. (2015) 13:147–53. doi: 10.1097/XEB.0000000000000054, 26317388

[11] Algarni M Hadi MA Yahyouche A Mahmood S Jalal Z. A mixed-methods systematic review of the prevalence, reasons, associated harms and risk-reduction interventions of over-the-counter (OTC) medicines misuse, abuse and dependence in adults. J Pharm Policy Pract. (2021) 14:76. doi: 10.1186/s40545-021-00350-7, 34517925 PMC8439034

[12] Brook I Yocum P Shah K. Aerobic and anaerobic bacteriology of concurrent chronic otitis media with effusion and chronic sinusitis in children. Arch Otolaryngol Head Neck Surg. (2000) 126:174–6. doi: 10.1001/archotol.126.2.174, 10680868

[13] Zhao AS Boyle S Butrymowicz A Engle RD Roberts JM Mouzakes J. Impact of 13-valent pneumococcal conjugate vaccine on otitis media bacteriology. Int J Pediatr Otorhinolaryngol. (2014) 78:499–503. doi: 10.1016/j.ijporl.2013.12.028, 24461461

[14] Caspary H Welch JC Lawson L Darrow D Buescher S Shahab S . Impact of pneumococcal polysaccharide vaccine (Prevnar) on middle ear fluid in children undergoing tympanostomy tube insertion. Laryngoscope. (2004) 114:975–80. doi: 10.1097/00005537-200406000-00004, 15179198

[15] Giebink GS Juhn SK Weber ML Le CT. The bacteriology and cytology of chronic otitis media with effusion. Pediatr Infect Dis. (1982) 1:98–103. doi: 10.1097/00006454-198203000-00007, 6983682

[16] Liston TE Foshee WS McCleskey FK. The bacteriology of recurrent otitis media and the effect of sulfisoxazole chemoprophylaxis. Pediatr Infect Dis. (1984) 3:20–4. doi: 10.1097/00006454-198401000-00006, 6366770

[17] Niedzielski A Chmielik LP Stankiewicz T. The formation of biofilm and bacteriology in otitis media with effusion in children: a prospective cross-sectional study. Int J Environ Res Public Health. (2021) 18:3555. doi: 10.3390/ijerph18073555, 33808050 PMC8037871

[18] Granath A Rynnel-Dagöö B Backheden M Lindberg K. Tube associated otorrhea in children with recurrent acute otitis media; results of a prospective randomized study on bacteriology and topical treatment with or without systemic antibiotics. Int J Pediatr Otorhinolaryngol. (2008) 72:1225–33. doi: 10.1016/j.ijporl.2008.04.015, 18565598

[19] Van Hoecke H De Paepe AS Lambert E Van Belleghem JD Cools P Van Simaey L . *Haemophilus influenzae* biofilm formation in chronic otitis media with effusion. Eur Arch Otorrinolaringol. (2016) 273:3553–60. doi: 10.1007/s00405-016-3958-9, 26946303

[20] Närkiö-Mäkelä M Meri S. Cytolytic complement activity in otitis media with effusion. Clin Exp Immunol. (2001) 124:369–76. doi: 10.1046/j.1365-2249.2001.01523.x, 11472396 PMC1906070

[21] Skovbjerg S Roos K Holm SE Grahn Håkansson E Nowrouzian F Ivarsson M . Spray bacteriotherapy decreases middle ear fluid in children with secretory otitis media. Arch Dis Child. (2009) 94:92–8. doi: 10.1136/adc.2008.137414, 18713796

[22] Bequignon E Dupuy L Zerah-Lancner F Bassinet L Honoré I Legendre M . Critical evaluation of Sinonasal disease in 64 adults with primary ciliary dyskinesia. J Clin Med. (2019) 8. doi: 10.3390/jcm8050619, 31067752 PMC6571605

[23] Sriwardhana KB Howard AJ Dunkin KT. Bacteriology of otitis media with effusion. J Laryngol Otol. (1989) 103:253–6. doi: 10.1017/S0022215100108631, 2495334

[24] Jero J Karma P. Bacteriological findings and persistence of middle ear effusion in otitis media with effusion. Acta Otolaryngol Suppl. (1997) 529:22–6. doi: 10.3109/00016489709124071, 9288259

[25] Närkiö-Mäkelä M Jero J Meri S. Complement activation and expression of membrane regulators in the middle ear mucosa in otitis media with effusion. Clin Exp Immunol. (1999) 116:401–9. doi: 10.1046/j.1365-2249.1999.00916.x, 10361226 PMC1905301

[26] Diamond C Sisson PR Kearns AM Ingham HR. Bacteriology of chronic otitis media with effusion. J Laryngol Otol. (1989) 103:369–71. doi: 10.1017/S0022215100108989, 2497219

[27] Bunse T Hildmann H Zan W Opferkuch W. A bacteriological study of otitis media with effusion. Concurrent coagulase-negative staphylococcal infections in the middle ear. Arch Otorhinolaryngol. (1987) 243:387–91. doi: 10.1007/BF00464648, 3566622

[28] Min HK Kim SH Park MJ Kim SS Kim SH Yeo SG. Bacteriology and resistance patterns of otitis media with effusion. Int J Pediatr Otorhinolaryngol. (2019) 127:109652. doi: 10.1016/j.ijporl.2019.109652, 31466026

[29] Kim SJ Chung JH Kang HM Yeo SG. Clinical bacteriology of recurrent otitis media with effusion. Acta Otolaryngol. (2013) 133:1133–41. doi: 10.3109/00016489.2013.816442, 24125184

[30] Jung H Lee SK Cha SH Byun JY Park MS Yeo SG. Current bacteriology of chronic otitis media with effusion: high rate of nosocomial infection and decreased antibiotic sensitivity. J Infect. (2009) 59:308–16. doi: 10.1016/j.jinf.2009.08.013, 19715725

[31] Kang DW Lee HJ Bang JH Kim SH Byun JY Park MS . Bacteriology and trends of antimicrobial resistance of Pseudomonas aeruginosa and methicillin-resistant *Staphylococcus aureus* in otitis media: a retrospective data analysis. Clin Otolaryngol. (2023) 48:872–80. doi: 10.1111/coa.14086, 37485597

[32] Lee JS Kim MG Hong SM Na SY Byun JY Park MS . Changing patterns of bacterial strains in adults and children with otitis media in korean tertiary care centers. Clin Exp Otorhinolaryngol. (2014) 7:79–86. doi: 10.3342/ceo.2014.7.2.79, 24917902 PMC4050092

[33] Xu Z Han C Dang Z Huang T Li Z Huang Y. The bacteriological characteristics and drug resistance of the nasopharyngeal region in 311 cases of adenoid hypertrophy with secretory otitis media. Lin Chung Er Bi Yan Hou Tou Jing Wai Ke Za Zhi. (2021) 35:428–31. 10.13201/j.issn.2096-7993.2021.05.010 PMC10128474 34304468

[34] Ungkanont K Jootakarn S Leelaporn A Kijsinthopchai U Tanphaichitr A Vathanophas V . Association between adenoid bacteriology and clinical characteristics of adenoid-related diseases in children. SAGE Open Med. (2021) 9:20503121211006005. doi: 10.1177/20503121211006005, 33868688 PMC8020219

[35] Thornton RB Wiertsema SP Kirkham L-AS Rigby PJ Vijayasekaran S Coates HL . Neutrophil extracellular traps and bacterial biofilms in middle ear effusion of children with recurrent acute otitis media--a potential treatment target. PLoS One. (2013) 8:e53837. doi: 10.1371/journal.pone.0053837, 23393551 PMC3564866

[36] Emami A Pirbonyeh N Moattari A Bazargani A Motamedifar M. Risk of otitis media with effusion (OME) in children by *Pseudomonas aeruginosa*. Int J Pediatr Otorhinolaryngol. (2019) 125:6–10. doi: 10.1016/j.ijporl.2019.06.017, 31229854

[37] Aliyeva A Sari E. Microbial profiles and antibiotic resistance in chronic Otorrhea: an evaluation of operated and non-operated patients. Indian J Otolaryngol Head Neck Surg. (2025) 77:3003–14. doi: 10.1007/s12070-025-05625-y, 40727155 PMC12297121

