# Supplementary material for: Global and temporal shifts in the cultured-based bacteriology of otitis media with effusion: a systematic review and meta-analysis
Source: Front Public Health. 2026 Oct 15;14:1909909. doi: 10.3389/fpubh.2026.1909909 (PMC13716855; doi:10.3389/fpubh.2026.1909909)
Supplement: Supplementary file 1 [file Supplementary_file_1.DOCX]

**Supplementary Table 1. Search strategy**

1. PubMed

("otitis media with effusion"[MeSH]) AND (“bacteria”[tiab] OR “bacteriology”[Mesh]) AND 1980:2025[dp]

2. Embase

("otitis media with effusion":ti,ab) AND (bacter*:ti,ab OR microbiolog*:ti,ab) AND [1980-2025]/py

3. Cochrane Library

("otitis media with effusion"):ti,ab,kw AND (bacteriolog* OR microbiolog*):ti,ab,kw

| Supplementary Table 2. Risk of bias for all included studies (JBI Tool) | | | | | | | | | | | | |
| --- | --- | --- | --- | --- | --- | --- | --- | --- | --- | --- | --- | --- |
| Study Author, Year | **Q1** | **Q2** | **Q3** | **Q4** | **Q5** | **Q6** | **Q7** | **Q8** | **Q9** | **Yes (n)** | **Yes (%)** | **Risk of Bias** |
| Brook I (2000) | No | Yes | No | Yes | Yes | Yes | Yes | Yes | Yes | 7 | 78% | Low |
| Zhao AS(2014) | No | Yes | No | Yes | Yes | Yes | Yes | Yes | Yes | 7 | 78% | Low |
| Caspary H. (2004) | Yes | Yes | Yes | Yes | Yes | Yes | Yes | Yes | Unclear | 8 | 89% | Low |
| Giebink (1982) | Yes | Unclear | Yes | Yes | No | Yes | Yes | Yes | Unclear | 6 | 67% | Low |
| Niedzielski (2021) | No | Yes | No | Yes | Yes | Yes | Yes | Yes | Yes | 7 | 78% | Low |
| Van Hoecke H (2016) | No | Yes | No | Yes | Yes | Yes | Yes | Yes | Yes | 7 | 78% | Low |
| Narkio-Makela M (2001) | Yes | Unclear | Yes | Yes | Yes | Yes | Yes | Yes | Unclear | 7 | 78% | Low |
| Skovbjerg S (2009) | No | Yes | No | Yes | Yes | Yes | Yes | Yes | Yes | 7 | 78% | Low |
| Sriwardhana (1989) | Yes | Yes | No | Yes | Yes | Yes | Yes | Yes | Unclear | 7 | 78% | Low |
| J. Jero (1997) | Yes | Yes | No | Yes | Yes | Yes | Yes | Yes | Unclear | 7 | 78% | Low |
| M. Nariko (1999) | Yes | Yes | No | Yes | Yes | Yes | Yes | No | Yes | 7 | 78% | Low |
| Diamond (1989) | Yes | Yes | No | Yes | Yes | Yes | Yes | No | Yes | 7 | 78% | Low |
| Bunse (1986) | No | Yes | No | Yes | Yes | Yes | Yes | Yes | Yes | 7 | 78% | Low |
| Min HK (2019) | No | Yes | No | Yes | Yes | Yes | Yes | Yes | Yes | 7 | 78% | Low |
| Kim SJ (2013) | No | Yes | No | Yes | Yes | Yes | Yes | Yes | Yes | 8 | 89% | Low |
| Jung H (2009) | No | Yes | No | Yes | Yes | Yes | Yes | Yes | Yes | 7 | 78% | Low |
| Kang DW (2023) | No | Yes | Yes | Yes | Yes | Yes | Yes | Yes | Yes | 8 | 89% | Low |
| Lee JS (2014) | No | Yes | No | Yes | Yes | Yes | Yes | Yes | Yes | 8 | 89% | Low |
| Xu Z (2021) | No | Yes | No | Yes | Yes | Yes | Yes | Yes | Yes | 8 | 89% | Low |
| Ungkanont K (2021) | Yes | Yes | Yes | Yes | Yes | Yes | Yes | Yes | Yes | 9 | 100% | Low |
| Thornton RB (2013) | Unclear | Yes | Yes | Yes | No | Yes | No | No | Unclear | 5 | 45% | Moderate |
| Emami A (2019) | Yes | Yes | Yes | Yes | Yes | Yes | Yes | Yes | Unclear | 8 | 89% | Low |

| **Supplementary Table 3. Funnel plots for culture-positivity and organism-specific pooled proportions before and 2000-onward** | | |
| --- | --- | --- |
| **Bacteriology** | **Pre-2000** | **2000-onward** |
| **Any growth** |  |  |
| ***H. influenzae*** |  |  |
| ***S. pneumoniae*** |  |  |
| **CoNS** |  |  |
| **MSSA** |  |  |
| ***M. catarrhalis*** |  |  |

| **Supplementary Table 4. Descriptive distribution of 1,926 reported bacterial isolate records from included studies** | | |
| --- | --- | --- |
| **Microbiology** | **No. of Isolates, n (%)** |  |
| *Coagulase-negative staphylococci* | 407 (21.1) |  |
| Methicillin-susceptible *S.aureus* | 329 (17.1) |  |
| *Haemophilus influenzae* | 328 (17.0) |  |
| *Streptococcus pneumoniae* | 250 (13.0) |  |
| Methicillin-resistant *S.aureus* | 228 (11.8) |  |
| *Moraxella catarrhalis* | 119 (6.2) |  |
| *Pseudomonas aeruginosa* | 90 (4.7) |  |
| ^a^Others | 175 (9.1) |  |
| Total | 1,926 (100) |  |

**Supplementary Table 5. Sensitivity analyses addressing the influence of Korean studies and potential cohort overlap**

| Outcome | Primary pooled estimate | After excluding all Korean studies | After de-overlapping Korean cohorts |
| --- | --- | --- | --- |
| Any growth | 0.434 (0.354, 0.518) | 0.558 (0.421, 0.687) | 0.706 (0.092, 0.983) |
| *H. influenzae* | 0.264 (0.177, 0.375) | 0.365 (0.305, 0.429) | 0.189 (0.015, 0.777) |
| CoNS | 0.261 (0.165, 0.387) | 0.179 (0.072, 0.379) | 0.299 (0.260, 0.341) |
| *S. pneumoniae* | 0.142 (0.108, 0.184) | 0.197 (0.146, 0.261) | 0.168 (0.065, 0.371) |
| MSSA | 0.160 (0.106, 0.234) | 0.154 (0.085, 0.263) | 0.356 (0.198, 0.552) |
| MRSA | 0.131 (0.079, 0.210) | 0.140 (0.060, 0.294) | 0.297 (0.100, 0.617) |
| *P. aeruginosa* | 0.086 (0.043, 0.165) | 0.175 (0.062, 0.405) | 0.079 (0.029, 0.195) |
| *M. catarrhalis* | 0.087 (0.041, 0.177) | 0.147 (0.084, 0.244) | 0.065 (0.012, 0.281) |
